# Genome sequence of the *Pseudomonas aeruginosa* bacteriophage Shea

**DOI:** 10.1128/mra.00086-26

**Published:** 2026-06-18

**Authors:** Iñigo Caballero Quiroga, Michael Loccisano, Aiden Stanciu, Delroy Brockett, Jonathan Luo, Sherin Kannoly, Olga Calderon, John J. Dennehy, Anna A. Feitzinger, Monica Trujillo, Fabrizio Spagnolo

**Affiliations:** 1 Biology Department, Queens College of The City University of New York, New York, New York, USA; 2 Biology Doctoral Program, The Graduate Center of The City University of New York 2009, New York, New York, USA; 3 Natural Sciences Department, LaGuardia Community College of The City University of New York 14777 , New York, New York, USA; 4 DNA Learning Center, Cold Spring Harbor Laboratory 2595, Cold Spring Harbor, New York, USA; 5 Department of Biological Sciences and Geology, Queensborough Community College of The City University of New York 14782 , New York, New York, USA; 6 Department of Natural and Life Sciences, Long Island University Post 42805 , New York, New York, USA; DOE Joint Genome Institute, Berkeley, California, USA

**Keywords:** pseudolysogeny, carrier state

## Abstract

Bacteriophage Shea, which infects the opportunistic pathogen *Pseudomonas aeruginosa*, likely has a non-canonical pseudolysogenic lifestyle. Here, we present its complete 43,333 bp genome sequence. This announcement will contribute to investigations of this underappreciated phage lifestyle.

## ANNOUNCEMENT

While most dsDNA bacteriophages are lytic or lysogenic, some exhibit pseudolysogeny, where the phage genome remains dormant without integrating into the host genome (1–3). These phage genomes can persist for multiple generations before either entering the lytic cycle or being lost, reverting the host to a phage-sensitive state. We sequenced the Shea genome to investigate its potential pseudolysogenic lifestyle.

**Fig 1:**
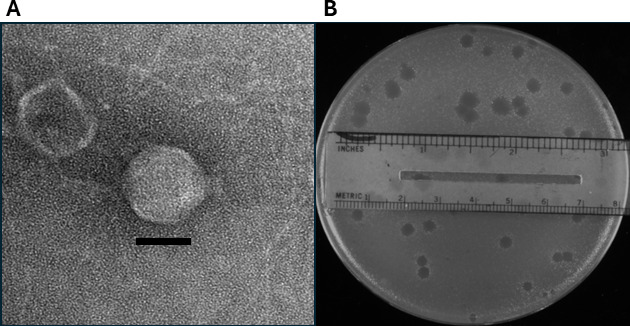
Phage Shea TEM image (**A**). Bacteriophage particles were negatively stained with 1% uranyl acetate on 200 mesh carbon–formvar-coated copper grid and viewed with a JEOL JEM 1400Flash electron microscope with 120 keV and 120 kV accelerating voltage and a magnification of 80,000×. Scale bar is 50 nm. Plaque morphology (**B**). Shea makes 3–5 mm turbid plaques on *P. aeruginosa* MPA01. Image acquired with a Biorad Gel Doc XR+ System.

For phage isolation, mud puddle water from Flushing, NYC (November 2017; 40.75539, −73.84935) was centrifuged (600 × *g*, 5 min) and 0.22 μm filtered. Filtrate (100 μL) and 100 μL exponential *P. aeruginosa* MPA01 were incubated in 10 mL lysogeny broth (18 h, 37°C). After re-filtration, the lysate was serially diluted and spot titered. A 3 mm turbid plaque was purified via three double-agar overlay rounds (Fig. 1) (4). For concentration, 10 mL of lysate was precipitated (20% PEG-8000/2.5 M NaCl) overnight at 4°C, centrifuged (19,000 × *g*), and resuspended in 1 mL SM buffer.

Genomic DNA was purified from 1 mL concentrated lysate using a Wizard DNA Extraction Kit (Promega). SeqCoast Genomics (Portsmouth, NH) performed library preparation (Illumina DNA Prep Tagmentation Kit with unique dual indexes) and 2 × 150 bp sequencing on an Illumina NextSeq 2000 using a 300 Cycle Flow Cell Kit, yielding 1,641,236 paired reads. Read demultiplexing, read trimming, and run analytics were performed via DRAGEN v3.10.12, and reads were assembled into a single contig (4,747× coverage) using SPAdes v4.2.0 (5).

Bacteriophage Shea (*Jamesmcgillvirus*, *Fredfastierviridae*) has a 43,333-bp genome (45.34% guanine + cytosine [G + C]) and podovirus morphology (Fig. 1). Nucleotide collection (nr/nt) BLAST identified *Pseudomonas* phage PaP2 (NC_005884.1) as the closest relative (OrthoANIu: 98.57% identity) (6). PhageTerm v1.0.12 indicated terminally redundant ends (7). Genome annotation via Pharokka v1.3.2 predicted 58 coding sequences (8). Aragorn v1.2.36 detected no tRNA genes (9).

Although Pharokka predicted no lysogeny-associated genes, Shea produced turbid plaques (Fig. 1). To further investigate, we performed three rounds of colony isolation of a microcolony picked from a turbid plaque. Clonal isolates showed superinfection resistance, yet recovered lysates still produced plaques on *P. aeruginosa*. Genomic DNA from an overnight culture was extracted via a Wizard kit. A sequencing library was prepared using the ONT Rapid Barcoding Kit 24 V14 (SQK-RBK114.24) with no shearing/size selection and sequenced on a MinION Mk1B with an R10.4.1 Flongle flow cell. High-accuracy basecalling (Dorado 7.8.3 with the dna_r10.4.1_e8.2_400bps_hac@v4.3.0. model) and demultiplexing (MinKNOW 25.05.14) yielded 12,606 reads (N50: 15,990 bp). Reads were filtered (NanoFilt v2.8.0; Q > 8, length >500 bp) and assembled (Flye 2.9.5-b1801, --nano-hq, 6.3 Mb expected size). All software were run with default parameters, except where noted. The assembly produced two contigs: a 6,276,556 bp *P. aeruginosa* genome and a 41,534 bp Shea genome. Minimap 2-2.30 (r1287) mapped all reads to either *P. aeruginosa* or Shea (10). The absence of chimeric Illumina reads mapping to both host and phage genomes and showing an integration site support the hypothesis that Shea does not integrate into the host chromosome and instead persists in an episomal state as a pseudolysogen.

## Data Availability

The genome sequence and associated data for phage Shea were deposited under GenBank accession number PX395730, BioProject number PRJNA1330251, BioSample number SAMN51482869, and SRA number SRR35438347. Raw reads generated from ONT sequencing of Shea-infected *P. aeruginosa* were deposited under BioProject number PRJNA1425019, BioSample number SAMN55402708, and SRA number SRR37263212.
